# Editorial: Methods and applications of natural language processing in psychiatry research

**DOI:** 10.3389/fpsyt.2022.972799

**Published:** 2022-07-11

**Authors:** Li Wang, Shuyan Li, Hui Chen, Yunyun Zhou

**Affiliations:** ^1^Research Center for Intelligence Information Technology, Nantong University, Nantong, China; ^2^School of Medical Information and Engineering, Xuzhou Medical University, Xuzhou, China; ^3^School of Biomedical Engineering, Capital Medical University, Beijing, China; ^4^Children's Hospital of Philadelphia, Philadelphia, PA, United States

**Keywords:** text mining, machine learning, natural language processing, psychiatry, information retrieval

Natural language processing (NLP), as a branch of Artificial Intelligent (AI) techniques, helps machines to understand unstructured human language. Nowadays, more and more NLP models have been used for text data mining to explore meaningful medical concepts from clinical notes and social media. For example, Wang et al. (1) extracted drug-drug interactions from AERS reports, Pham et al. (2) predicted health care trajectories from medical records, Mukherje et al. (3) used quantification method to evaluate the psychiatric patients' mental status. In this Research Topic, all the accepted manuscripts have been rigorously peer-reviewed by researchers who have strong computational psychiatry research background. The innovative applications of these projects will improve the efficiency, effectiveness, and quality of data processing in diagnosis and treatments for mental health.

Among these accepted manuscripts, two of them used online question answering dataset to address mental health problems. The social question answering based online counseling (SQA-OC) dataset used for people seeking professional mental health information and service, has become a crucial pre-consultation and application stage toward online counseling. In SQA-OC, public's perception of counselors' service quality used the notion of perceived helpfulness to evaluate a counselor's service. However there is no such a tool to facilitate the explanation of online questioning and answering (QA) for the quality of mental health service efficiently. Huang et al. developed a computational linguistic and explainable machine learning predictive model to explore how various sources and types of linguistic cues contributed to the perceived helpfulness. Online inquiry platforms, which is the place for people asking questions anonymously, have become important information sources for those people who are concerned about social stigma and discrimination for their mental disorders. Therefore, examining people's query for mental disorders would be useful for designing educational programs for communities. Park et al. developed computational methods to examine the contents of the queries regarding mental disorders that posted on online inquiry platforms.

Another two interesting projects discussed the impact of psychiatric disorders suffering from other diseases on patients. Mental disorder of people living with HIV (PLWH) has become an emerging worldwide public health issues. Wang et al. explored the relationship between anxiety, depression, and sleep disorders for PLWH through a network approach. The network model featured 28 symptoms for 4,091 HIV-infected individuals. Node predictability and strength centrality were utilized to assess the importance of items. Authors estimated and compared 20 different networks based on subpopulations of males and females to analyze similarities and differences in a network structure, connections, and symptoms. Another type of anxiety is observed in patients with systemic lupus erythematosus (SLE). In this study, to recognize abnormal T-cell and B-cell subsets of SLE patients with anxiety, patient's disease phenotypes data from electronic lupus symptom records were extracted by NLP tool. The Hospital Anxiety and Depression Scale was used to distinguish 107 patients who were selected to meet research requirements. The final findings not only find the difference of T-cell subsets in SLE patients with or without anxiety, but also imply that T cells might play an important role in patients with anxiety disorder (Gu et al.).

Almost all the accepted articles took use of machine learning or deep learning methods, especially in these following two works. Xie et al. used Relevance Vector Machine (RVM) to increase generalizability and clinical interpretability of classifiers. It is a typical sparse Bayesian classifier less prone to overfitting with small training datasets. RVM was optimized by leveraging automatic recursive feature elimination and expert feature refinement from the perspective of health linguistics. Authors evaluated the diagnostic utility of the Bayesian classifier under different probability cut-offs in terms of sensitivity, specificity, positive and negative likelihood ratios against clinical thresholds for diagnostic tests, furthermore, illustrated interpretation of RVM tool in clinic using Bayes' nomogram. Mood disorders are ubiquitous mental disorders with familial aggregation. Wan et al. extracted family history of psychiatric disorders from large electronic hospitalization records in the past 10 years. Authors proposed a pre-trained language model [Bidirectional Encoder Representations from Transformers (BERT)-Convolutional Neural Network (CNN)]. They first project the electronic hospitalization records into a low-dimensional dense matrix *via* the pre-trained Chinese BERT model, then feed the dense matrix into the stacked CNN layer to capture high-level features of texts; finally, they used the fully connected layer to extract family history based on high-level features. They further studied the correlation between mood disorders and family history of psychiatric disorder.

In conclusion, this Research Topic mainly focused on the NLP application in the psychiatry area, some works took use of the NLP tools or methods directly, and other works modified the NLP algorithm. These work opens new possibilities for how technologies can help identify and predict psychiatric illness in patients. Finally, we sincerely thank all the authors who contributed their work and provided articles, allowing us to coordinate and edit this outstanding collection.

## Author contributions

All authors listed have made a substantial, direct, and intellectual contribution to the work and approved it for publication.

## Funding

This work was funded by National Science Foundation of China, No. 81873915.

## Conflict of interest

The authors declare that the research was conducted in the absence of any commercial or financial relationships that could be construed as a potential conflict of interest.

## Publisher's note

All claims expressed in this article are solely those of the authors and do not necessarily represent those of their affiliated organizations, or those of the publisher, the editors and the reviewers. Any product that may be evaluated in this article, or claim that may be made by its manufacturer, is not guaranteed or endorsed by the publisher.
